# Supplementary material for: The Challenging Life of Mutators: How Pseudomonas aeruginosa Survives between Persistence and Evolution in Cystic Fibrosis Lung
Source: Microorganisms. 2024 Oct 11;12(10):2051. doi: 10.3390/microorganisms12102051 (PMC11509988; doi:10.3390/microorganisms12102051)
Supplement: Supplementary file 1 [file microorganisms-12-02051-s001.zip › Supplementary_Table 2.pdf]

Table S2. Characteristics of the 70 *Pseudomonas aeruginosa* mutator strains isolated in 29 cystic fibrosis patients.

Patient ID	Isolate ID	Phenotype	Isolation year	ST	SNPs vs ancestor	Putative mutation	Mutation summary	Resistome mutation
**2**	PA_02_04	PAM	2013	395	189			21
**2**	PA_02_06	PAM	2015	703	259			24
**2**	PA_02_10	PA MDR	2019	703	1573	mutT P136S;E236D	mutT	26
**2**	PA_02_11	PA MDR	2019	703	1018	mutT P136S;E236D	mutT	31
**3**	PA_03_13	PA MDR	2018	395‡	317	mutS D510G	mutS	24
**4**	PA_04_03	PA	2012	260	101			24
**5**	PA_05_13	PAM MDR	2022	274	224			22
**7**	PA_07_02	PA	2010	274	216			24
**7**	PA_07_04	PA MDR	2012	274	545			26
**7**	PA_07_05	PA ColR	2012	274	480			25
**7**	PA_07_07†	PA MDR CoR	2014	274	614			25
**7**	PA_07_11	PA XDR	2016	4903	506			26
**7**	PA_07_12	PA MDR CoR	2018	274	754			26
**7**	PA_07_14	PA MDR	2020	274	874			26
**9**	PA_09_05	PAM XDR	2011	262	284	mutS Y488*	mutS	29
**9**	PA_09_09	PAM XDR	2015	262	460	mutS Y488*	mutS	29
**9**	PA_09_15	PA XDR	2019	3243	262			28
**11**	PA_11_07	PA MDR	2018	4905	213	mutS W307*	mutS	39
**11**	PA_11_14	PA XDR	2021	4905	513	mutS W307*	mutS	38
**12**	PA_12_07	PA	2014	934	238			23
**12**	PA_12_09	PAM MDR	2015	934	284			22
**12**	PA_12_13	PA MDR	2017	934	632			23
**12**	PA_12_16	PA XDR	2019	934	813	mutT G303S + pfpI A118V	combination	28
**14§**	PA_14_02	PAM MDR CoR	2012	439	311			31
**14§**	PA_14_03	PA MDR CoR	2012	439	475			31
**14§**	PA_14_07	PA MDR	2016	439	1458	mutS S647F + mutT H299Y + ung Y73C	combination	27

**14**[§]	PA_14_15	PA XDR	2020	439	1635	mutS S647F + mutT H299Y + ung Y73C	combination	29
**15**	PA_15_02	PA	2012	245	116			28
**15**	PA_15_03	PA MDR	2012	245	156			27
**15**	PA_15_04	PAM MDR	2012	245	165			28
**15**	PA_15_05	PA	2012	245	123			27
**15**	PA_15_11	PAM XDR CoR	2018	245	386	mutS 1713_1728del, D605*	mutS	28
**15**	PA_15_20	PAM MDR	2022	245	482	mutS 1713_1728del, D605*	mutS	30
**15**	PA_15_21	PAM XDR	2022	245	431	mutS 1713_1728del, D605*	mutS	30
**17**	PA_17_02	PAM MDR	2012	274	128	mutL Q135*	mutL	23
**18**	PA_18_09	PA MDR	2017	621	334	ung E156*	ung	26
**20**[§]	PA_20_04	PA XDR	2013	3243	320	mutS D702N	mutS	27
**20**[§]	PA_20_07	PA MDR	2015	3243	456	mutS D702N	mutS	26
**20**[§]	PA_20_08	PA XDR	2017	3243	571	mutS D702N	mutS	28
**21**[§]	PA_21_5	PAM XDR	2018	274	62	mutL Q135*	mutL	24
**21**[§]	PA_21_7	PAM XDR	2019	274	118	mutL Q135*	mutL	24
**23**[§]	PA_23_05	PAM MDR	2014	242	508	mutS 1537_1555del17bp, G163*	mutS	26
**25**	PA_25_03	PA MDR	2005	4946	135			34
**25**	PA_25_04	PA XDR	2007	4946	394	radA T36I	radA	34
**25**	PA_25_05[†]	PA XDR	2008	4946	484	radA T36I	radA	34
**25**	PA_25_09	PA MDR	2012	253[‡]	203	mutS M673*	mutS	29
**27**	PA_27_03	PAM XDR	2010	4950	208	mutL Q135*	mutL	22
**27**	PA_27_06	PAM XDR	2014	4950	351	mutL Q135*	mutL	23
**28**	PA_28_02	PAM	2012	676	625	mutY R105C	mutY	27
**28**	PA_28_03	PA MDR	2012	676	594	mutS N563* + mutY E97G; R105C	combination	29
**28**	PA_28_04[†]	PAM	2014	676	1328	mutS N563*	mutS	28
**28**	PA_28_09	PA MDR CoR	2017	676	1430	mutY R105C	mutY	29
**28**	PA_28_11	PA MDR	2019	4951[‡]	720	mutT H28R; E236D	mutT	26
**29**	PA_29_09[†]	PA MDR	2021	3243[‡]	416	mutS Q480*	mutS	30
**32**	PA_32_15	PAM	2020	262	429	mutS W307*	mutS	20
**34**	PA_34_10	PA	2018	309[‡]	280	uvrD K344R; G434S; V556I; A641S; S662N; N666S	uvrD	31
**38**	PA_38_02	PAM MDR	2011	4954	303	mutL R633*	mutL	37
**38**	PA_38_03	PA MDR CoR	2013	4954	684	mutL R633*	mutL	31
**40**	PA_40_02	PA MDR	2013	3243	217	mutL G42D; T147A	mutL	25

**40**	PA_40_04	PA MDR	2017	3243	186			28
**40**	PA_40_06	PA MDR	2019	3243	488	mutL G42D; T147A + uvrD G150E; R621Q	combination	29
**43**§	PA_43_03	PA MDR	2008	3243	158			26
**43**§	PA_43_04	PA MDR	2008	3243	149			27
**44**§	PA_44_02	PA MDR	2009	830	170			33
**48**§	PA_48_03	PA XDR	2007	4956	229			26
**49**§	PA_49_03	PA	2011	560	80			27
**49**§	PA_49_05	PA XDR	2013	560	321	mutL A406T; E506*	mutL	27
**50**§	PA_50_01†	PAM MDR	2010	4958	795			20
**50**§	PA_50_03	PA MDR	2010	4958	2215	mutL S486G + mutS G618S + pfpI G53D + uvrD V188A; F326L	combination	31
**51**	PA_51_03†	PA MDR	2010	3243‡	151	mutS T493P	mutS	28

For each strain, the identified putative mutations causing hypermutability and the total number of mutations in the resistome genes are reported.

ST, sequence type; SNPs, single nucleotide polymorphisms; PA, *Pseudomonas aeruginosa*; PAM, mucoid *Pseudomonas aeruginosa*; MDR, multi drug resistant; XDR, extensively drug resistant; CoR, Colistin resistant; § indicates patients with poor prognosis (dead/transplanted); †indicates strains that has evolved in a new ST; ‡ indicates ST that has reverted to the non-mutator state by losing the mutation; * indicates a stop mutation
