# Supplementary material for: The Challenging Life of Mutators: How Pseudomonas aeruginosa Survives between Persistence and Evolution in Cystic Fibrosis Lung
Source: Microorganisms. 2024 Oct 11;12(10):2051. doi: 10.3390/microorganisms12102051 (PMC11509988; doi:10.3390/microorganisms12102051)
Supplement: Supplementary file 1 [file microorganisms-12-02051-s001.zip › Supplementary_Table 1.pdf]

Table S1. Phenotypic resistance of the 70 *Pseudomonas aeruginosa* mutator strains. Antibiotics susceptibility expressed by Minimum Inhibitory Concentration (MIC)

Isolate ID	Phenotype	Mer	Ak	Azt	Cp	P/T	C/T	Co	Caz	CZA	Imp	To	Cpe	Lvx
PA_02_04	PAM	<=1	<4	<=2	<=0.25	<=4	<0.5	<=2	<=4	<=2	<=1	<=1	<=1	<=0.5
PA_02_06	PAM	<=1	16	4	>2	16	1	2	4	2	1	2	8	>4
PA_02_10	PA MDR	2	>16	<=2	>2	<=4	2	2	4	2	16	>16	>32	>4
PA_02_11	PA MDR	16	<4	16	>2	>64	2	<=2	>64	8	8	<=1	>32	>32
PA_03_13	PA MDR	>16	<4	16	1	16	>8	<=2	>64	>32	>16	>16	>32	3
PA_04_03	PA	<=1	>16	<=2	1	<=4	2	<=2	<=4	2	1	>16	>32	3
PA_05_13	PAM MDR	8	>16	8	>2	>64	1	<=2	16	2	8	8	>32	>4
PA_07_02	PA	2	>16	<=2	>2	4	2	<=2	4	4	>16	4	8	12
PA_07_04	PA MDR	2	>16	>16	>2	>64	>8	<=2	>64	2	8	>16	>32	6
PA_07_05	PA CoR	<=1	>16	<=2	2	<=4	<0.5	>4	<=4	<=2	4	>16	6	3
PA_07_07	PA MDR CoR	>16	>16	8	2	8	1	>4	<=4	<=2	>16	4	>32	12
PA_07_11	PA XDR	>16	>16	>16	>2	>64	>8	2	>64	4	>16	8	>32	16
PA_07_12	PA MDR CoR	8	8	8	2	16	0.5	>4	<=4	<=2	16	<=1	>32	4
PA_07_14	PA MDR	16	>16	8	>2	>64	4	<=2	16	2	>16	2	>32	>4
PA_09_05	PAM XDR	16	>16	>16	>2	>64	>8	<=2	>64	8	>16	8	>32	>4
PA_09_09	PAM XDR	8	>16	>16	>2	>64	>8	<=2	>64	16	>16	4	>32	>4
PA_09_15	PA XDR	>16	>16	>16	>2	>64	>8	2	>64	32	>16	>16	>32	3
PA_11_07	PA MDR	1	8	>16	2	>64	>8	<=2	>64	8	<=1	<=1	>32	16
PA_11_14	PA XDR	16	>16	>16	>2	>64	>8	2	>64	8	>16	2	>32	>4
PA_12_07	PA	<=1	>16	<=2	>2	<=4	1	<=2	<=4	<=2	1	>16	>32	12
PA_12_09	PAM MDR	1	>16	>16	>2	32	4	<=2	16	2	4	>16	>32	>4
PA_12_13	PA MDR	1	>16	>16	>2	>64	4	2	>64	4	4	>16	>32	>4
PA_12_16	PA XDR	>16	>16	>16	>2	>64	>8	<=2	>64	>32	>16	8	>32	>4
PA_14_02	PAM MDR CoR	8	>16	>16	>2	16	>8	>4	>64	<=2	8	8	>32	>4
PA_14_03	PA MDR CoR	4	>16	16	>2	16	4	>4	>64	<=2	8	>16	>32	>4

PA_14_07	PA MDR	16	<4	>16	2	>64	2	<=2	>64	8	8	<=1	>32	>4
PA_14_15	PA XDR	>16	16	>16	>2	>64	>8	<=2	>64	>32	8	2	>32	>4
PA_15_02	PA	8	>16	8	0.25	8	1	<=2	<=4	<=2	16	>16	>32	<=0.5
PA_15_03	PA MDR	8	>16	2	1	>64	2	<=2	8	2	16	8	>32	0.75
PA_15_04	PAM MDR	8	>16	>16	2	>64	4	<=2	>64	8	4	8	>32	>4
PA_15_05	PA	2	8	2	0.25	<=4	<0.5	<=2	<=4	<=2	2	2	>32	4
PA_15_11	PAM XDR CoR	8	>16	>16	>2	>64	>8	>4	>64	8	>16	8	>32	>4
PA_15_20	PAM MDR	>16	>16	>16	>2	>64	4	<=2	8	4	16	8	>32	>4
PA_15_21	PAM XDR	16	>16	>16	>2	>64	>8	<=2	>64	>32	8	4	>32	6
PA_17_02	PAM MDR	8	>16	>16	1	>64	>8	<=2	>64	8	8	>16	>32	2
PA_18_09	PA MDR	16	>16	2	>2	8	8	<=2	>64	16	>16	>16	>32	>4
PA_20_04	PA XDR	16	16	>16	2	>64	8	<=2	>64	16	>16	<=1	>32	>4
PA_20_07	PA MDR	16	>16	16	2	>64	4	<=2	>64	8	>16	4	>32	8
PA_20_08	PA XDR	16	>16	>16	>2	>64	4	<=2	>64	8	>16	4	>32	>4
PA_21_5	PAM XDR	16	>16	>16	2	>64	>8	<=2	>64	16	16	8	>32	8
PA_21_7	PAM XDR	4	>16	>16	2	>64	>8	<=2	>64	>32	1	8	>32	4
PA_23_05	PAM MDR	>16	16	16	>2	>64	2	<=2	>64	8	>16	<=1	>32	>4
PA_25_03	PA MDR	16	>16	4	>2	<=4	1	<=2	8	<=2	>16	>16	>32	>4
PA_25_04	PA XDR	32	>16	>16	>2	>16	>8	<=2	>64	>32	>16	>16	>32	>4
PA_25_05	PA XDR	32	>16	>16	>2	>16	>8	<=2	>64	>32	>16	>16	>32	>4
PA_25_09	PA MDR	16	16	>16	1	32	1	<=2	8	<=2	8	2	8	>4
PA_27_03	PAM XDR	16	>16	>16	>2	>16	>8	<=2	>64	>32	>16	>16	>32	>4
PA_27_06	PAM XDR	16	>16	>16	>2	>16	4	<=2	>64	4	>16	>16	>32	>4
PA_28_02	PAM	<=1	>16	<=2	0.25	<=4	<=0.5	<=2	<=4	<=2	<=1	>16	8	<=0.5
PA_28_03	PA MDR	8	>16	2	0.5	>16	<=0.5	<=2	<=4	<=2	>16	>16	>32	<=0.5
PA_28_04	PAM	1	>16	<=2	1	<=4	1	<=2	<=4	<=2	4	>16	>32	>1
PA_28_09	PA MDR CoR	>32	>16	16	>2	16	4	>4	>64	<=2	>16	>16	>32	>4
PA_28_11	PA MDR	32	>16	<=2	>2	<=4	1	4	<=4	<=2	>16	>16	>32	>4
PA_29_09	PA MDR	2	16	<=2	0.5	<=4	<=0.5	<=2	<=4	<=2	<=1	<=1	<=1	>1

PA_32_15	PAM	4	>16	2	1	8	<=0.5	<=2	4	<=2	>16	<=1	8	>4
PA_34_10	PA	1	>16	<=2	>2	<=4	<=0.5	<=2	<=4	<=2	>16	>16	8	>4
PA_38_02	PAM MDR	4	>16	>16	>2	16	4	<=2	32	4	>16	>16	>32	>4
PA_38_03	PA MDR CoR	4	>16	16	>2	>16	2	>4	32	<=2	>16	>16	>32	>4
PA_40_02	PA MDR	32	>16	16	1	<=4	>8	<=2	>64	8	>16	>16	>32	>4
PA_40_04	PA MDR	32	>16	<=2	>2	<=4	>8	<=2	32	8	>16	>16	>32	>4
PA_40_06	PA MDR	4	>16	<=2	>2	<=4	2	<=2	>64	<=2	>16	>16	>32	>4
PA_43_03	PA MDR	16	>16	16	>2	<=4	4	<=2	>64	4	>16	>16	>32	>4
PA_43_04	PA MDR	16	>16	>16	>2	>16	>8	<=2	>64	8	>16	>16	>32	>4
PA_44_02	PA MDR	8	16	>16	>2	>16	4	<=2	32	<=2	>16	>16	>32	>4
PA_48_03	PA XDR	32	>16	>16	>2	>16	>8	<=2	>64	>32	>16	>16	>32	>4
PA_49_03	PA	1	>16	16	>2	<=4	1	<=2	<=4	<=2	>16	>16	8	>4
PA_49_05	PA XDR	>32	>16	>16	0.5	>16	>8	<=2	>64	>32	>16	>16	>32	>4
PA_50_01	PAM MDR	4	>16	4	>2	<=4	2	<=2	4	<=2	>16	4	>32	>4
PA_50_03	PA MDR	4	<=4	>16	>2	>16	1	<=2	8	<=2	>16	<=1	>32	<=0.5
PA_51_03	PA MDR	32	>16	>16	>2	8	>8	<=2	32	4	>16	>16	>32	>4

According to EUCAST, colours represent three susceptibility categories: Red, Resistant; Yellow, Susceptible, increased exposure; Green, Susceptible, standard dosing regimen. For aminoglycosides, no susceptibility categories were assigned due to the absence of EUCAST breakpoints for pulmonary infections. Mer, Meropenem; Ak, Amikacin; Azt, Aztreonam; Cp, Ciprofloxacin; P/T, Piperacillin/Tazobactam; C/T, Ceftolozane/Tazobactam; Co, Colistin; Caz, Ceftazidime; CZA, Ceftazidime/Avibactam; Imp, Imipenem; To, Tobramycin; Cpe, Cefepime; Lvx, Levofloxacin; PA, *Pseudomonas aeruginosa*; PAM, mucoid *Pseudomonas aeruginosa*; MDR, multi drug resistant; XDR, extensively drug resistant; CoR, Colistin resistant.
